# Congenital/Inherited CD59 Deficiency: A Systematic Review

**DOI:** 10.3390/jcm15197751

**Published:** 2026-10-08

**Authors:** Lama Alwahiby, Lanah Alsuhim, Manal Alzahrani, Jalnar Albaloshi, Cristina Skrypnyk, Kannan Sridharan

**Affiliations:** 1 College of Medicine and Health Sciences, Arabian Gulf University, Manama P.O. Box 26671, Bahrain; lamaeaw@agu.edu.bh (L.A.); lanahass@agu.edu.bh (L.A.); manaldaz@agu.edu.bh (M.A.); jalnarasb@agu.edu.bh (J.A.); 2 Department of Molecular Medicine, AlJawhara Center, College of Medicine and Health Sciences, Arabian Gulf University, Manama P.O. Box 26671, Bahrain; cristinas@agu.edu.bh; 3 Department of Pharmacology and Therapeutics, College of Medicine and Health Sciences, Arabian Gulf University, Manama P.O. Box 26671, Bahrain

**Keywords:** systematic review, congenital CD59 deficiency, membrane attack complex, hemolytic anemia, polyneuropathies, eculizumab

## Abstract

**Background:** Inherited CD59 deficiency is a rare autosomal recessive complement-mediated disorder caused by biallelic loss-of-function variants in the *CD59* gene. It typically presents in infancy with Coombs-negative hemolytic anemia, relapsing demyelinating polyneuropathy, and recurrent ischemic strokes. Diagnosis is often delayed due to overlapping with more common neurological and hematological disorders. The present study was carried out to systematically review the literature on congenital CD59 deficiency, focusing on genotype–phenotype correlations, geographic distribution, clinical presentation, diagnostic approaches, and treatment outcomes. **Methods:** Eligible studies included case reports and case series of genetically or functionally confirmed inherited CD59 deficiency. Demographic, molecular, clinical, and therapeutic data were extracted and analyzed descriptively. **Results:** 20 reports included. Evidence remains mainly limited to case reports and small case series. The disorder demonstrates a severe and heterogeneous phenotype with predominant neuromuscular involvement, frequently associated with hematologic and cerebrovascular manifestations. Most reported cases originate from regions with high consanguinity, particularly the Middle East and Asia, suggesting possible founder effects and underrecognized prevalence. Exploratory analysis suggested a possible relationship between predicted variant functional impact and clinical severity. However, given the small and heterogeneous evidence base, this finding should be considered hypothesis-generating. Responses to conventional immunomodulatory therapies are variable and often transient. Eculizumab has shown clinical benefit in some patients, although treatment responses remain inconsistent. **Conclusions:** Congenital CD59 deficiency is a severe and underrecognized complement-mediated disorder with major diagnostic and therapeutic challenges. Current evidence remains insufficient to establish definitive genotype–phenotype correlations or standardized management strategies. Increased clinical awareness, early integration of functional and genetic testing, and further studies are needed to improve diagnosis and optimize treatment outcomes.

## 1. Introduction

The *CD59* gene is located on chromosome 11p13 and encodes a small glycosylphosphatidylinositol (GPI)-anchored membrane protein, protectin, which blocks formation of the membrane attack complex (MAC) by preventing C9 polymerization and provides protection to host cells from complement-mediated lysis [1]. Loss of CD59 function results in increased susceptibility of erythrocytes, peripheral nerves, and endothelial tissues to complement-mediated lysis and inflammation [2]. Two forms of CD59 deficiency are recognized: (1) secondary/acquired CD59 deficiency, such as in paroxysmal nocturnal hemoglobinuria (PNH) resulting from PIGA somatic defects; and (2) congenital/inherited CD59 deficiency, an exceptionally rare inborn error of immunity with distinct neurologic and hematologic manifestations [3].

Congenital or inherited CD59 deficiency (OMIM #612300; ORPHA:169464, ICD-11:4A00.1Y) is an ultra-rare autosomal recessive complement regulatory disorder caused by biallelic loss-of-function variants in the *CD59* gene. A spectrum of pathogenic variants, including missense, nonsense, and splice-site mutations, has been described, with important effects on CD59 expression and complement regulation [2]. Recognized as one of the most devastating complement-mediated disorders, it typically presents in infancy with recurrent Coombs-negative hemolytic anemia, relapsing demyelinating polyneuropathy resembling Guillain-Barré syndrome (GBS) or chronic inflammatory demyelinating polyneuropathy (CIDP), and recurrent ischemic strokes [4]. Although the global number of genetically confirmed patients remains limited, reports from Gulf-region populations have suggested that consanguinity and founder variants may contribute to local disease burden [5,6,7,8].

The clinical phenotype is characterized by hematologic, neurologic, and cerebrovascular involvement. Hematologically, patients typically present with chronic intravascular hemolysis and absent CD59 expression on red blood cells by flow cytometry [9]. Neurologic manifestations include recurrent early-onset demyelinating neuropathy resembling GBS or CIDP, sometimes progressing to persistent neurological deficits [10]. Cerebrovascular complications include ischemic stroke and retinal ischemia [11], while optic neuritis and Moyamoya syndrome have also been reported in rare cases [12,13]. Additional manifestations of immune dysregulation and atypical phenotypes have been occasionally described [14]. A summary of reported phenotype is presented in Table S1.

Diagnosis is often delayed because of phenotypic overlap with more common neurohematologic disorders, contributing to irreversible neurological disability and significant morbidity [5,6]. Markedly reduced or absent CD59 expression on blood cells by flow cytometry represents a key diagnostic clue, while definitive confirmation requires germline molecular testing demonstrating biallelic pathogenic variants in CD59 [1].

Therapeutic evidence remains limited. Conventional immunomodulatory therapies, including intravenous immunoglobulin, corticosteroids, and plasmapheresis, may provide transient benefit but are frequently associated with relapse [5]. Targeted complement inhibition with eculizumab has emerged as a disease-specific therapeutic strategy and has demonstrated marked hematologic and neurologic improvement in selected patients by preventing terminal complement activation and MAC formation [7,15]. However, long-term outcome data remain scarce, and genotype–phenotype relationships, natural history, and predictors of therapeutic response remain incompletely defined [16].

The literature to date consists primarily of case reports, small case series, mechanistic studies, and early treatment experiences. To our knowledge, this represents the first systematic review to comprehensively synthesize all reported patients, integrating molecular, clinical, therapeutic, and geographic data to define genotype–phenotype correlations and clinical outcomes, improve early recognition, guide therapeutic decision-making, and inform genetic counseling in affected populations.

## 2. Methodology

### 2.1. Study Design

This systematic review was designed in accordance with the Preferred Reporting Items for Systematic Reviews and Meta-Analyses (PRISMA) 2020 guidelines. The review protocol was registered on the Open Science Framework (Registration Id: osf.io/fnm72).

### 2.2. Eligibility Criteria

Eligibility criteria were defined according to the population, exposure, comparator, and study design framework. The population included patients of any age with a confirmed diagnosis of congenital/inherited CD59 deficiency, defined by either (a) biallelic pathogenic/likely pathogenic variants in the *CD59* gene identified via germline genetic testing and/or (b) absent CD59 expression on erythrocytes/leukocytes confirmed by flow cytometry in a clinical context consistent with the disease. Secondary CD59 deficiency (such as PNH due to PIGA mutation) was excluded unless congenital, non-germline somatic cases were clearly distinguishable. Intervention/exposure is not applicable; no primary intervention was required for study inclusion due to the observational review of a genetic disease. For the treatment outcomes, exposure included any reported therapy (e.g., eculizumab, IVIG, steroids, plasmapheresis). Where available, comparisons were made between different therapeutic interventions and pre- and post-treatment clinical status within individual patients as reported. Considering the rarity of the literature on this topic, all published and unpublished case reports, case series, observational cohort studies, clinical trials and conference abstracts describing patients with congenital CD59 deficiency were included. Case series were defined as studies reporting ≥2 patients. Reviews, editorials, non-English, in vitro or cell-line, and animal studies were excluded. Reference lists of excluded reviews were screened to identify additional eligible primary studies.

### 2.3. Information Sources

A comprehensive and systematic literature search of all the available literature was conducted across PubMed/MEDLINE and Embase. The grey literature was also explored through conference abstract databases to minimize publication bias. The final search was performed on 19 January of 2026, with no restrictions on publication year, geographic location, or publication type. This study is reported in compliance with PRISMA checklist (Table S2).

### 2.4. Search Strategy

Search strategies combined vocabulary terms with free-text keywords related to congenital CD59 deficiency. Search terms included a combination of MeSH terms, Emtree terms, and free-text keywords including but not limited to “CD59 deficiency” OR “congenital CD59 deficiency” OR “inherited CD59 deficiency” OR “*CD59* gene”. The full electronic search strategy for each database is provided in the Supplementary Material (Table S3). Search strategies were adapted to each database. The search strategy incorporated disease terminology and gene nomenclature obtained from the OMIM database [17].

### 2.5. Study Selection Process

All retrieved citations were imported into Microsoft Excel, and duplicate records were removed. Two independent reviewers screened titles and abstracts using the predefined eligibility criteria. Full-text screening was subsequently performed for potentially eligible studies. Conference abstracts were screened separately using identical eligibility criteria. Disagreements were resolved through discussion, with interposition by a third reviewer when consensus was not reached.

### 2.6. Data Collection Process

Data extraction was performed using a standardized data extraction form developed prior to study initiation. Two reviewers independently extracted study and patient-level data. All reported clinical, genetic, and therapeutic outcomes were extracted regardless of follow-up duration or outcome measurement method. When unavailable, data were recorded as “Not reported” and corresponding authors were not contacted. Extracted variables included:

- Demographics and geographic origin.
- Genetic variant and mutation classification.
- Clinical phenotype and disease course.
- Neuroimaging findings.
- Laboratory and diagnostic findings.
- Treatment exposure and clinical response.
- Functional outcomes at last follow-up.

### 2.7. Standardization of Variables

To address heterogeneity in reporting across included studies, selected clinical variables were standardized into predefined categories prior to synthesis. Functional outcome at last follow-up was classified into improving (documented neurological recovery or functional improvement), stable (persistent deficits without progression), progressive (worsening neurological impairment), or severe disability (e.g., persistent vegetative state or complete paralysis). Disease course was additionally categorized as monophasic, relapsing-remitting, or chronic progressive based on the clinical trajectory described in each report. Neuroimaging findings were extracted verbatim and subsequently categorized into normal versus abnormal imaging, dominant radiological pattern (demyelinating, ischemic/vascular, atrophic, inflammatory, or mixed), and anatomical distribution (supratentorial, infratentorial, spinal, or multifocal). Age at symptom onset was extracted verbatim and categorized according to American Academy of Pediatrics developmental groupings into infancy (<2 years), childhood (≥2 to <12 years), and adolescence (≥12 to 21 years). Diagnostic recognition was then assessed by comparing the age of onset and diagnosis categories. Early recognition was defined as diagnosis occurring within the same life stage as symptom onset while delayed recognition was defined as a diagnosis at a later stage. As for postmortem diagnosis, it was classified as missed diagnosis. This classification is aligned with established definitions of delayed recognition based on the interval between onset of symptoms and diagnosis/symptom onset and diagnosis [18]. Because no validated CD59 deficiency-specific severity scale was identified, an operational definition of severe phenotype was used for descriptive purposes. This classification was applied retrospectively to the clinical manifestations reported in the included publications and was not intended to represent a validated measure of disease severity. The classification was used solely to facilitate structured description of the reported phenotype and exploratory comparison across variant groups.

### 2.8. Risk of Bias Assessment

Risk of bias was assessed using the Joanna Briggs Institute (JBI) critical appraisal tools for case reports and case series. Each checklist item scored as 1 for “Yes” or 0 for “No/Unclear/Not applicable”. For descriptive purposes, JBI appraisal results were summarized as the number of fulfilled JBI criteria for each study. Total scores were converted into percentages and used to determine the risk of bias. Studies were categorized as high risk (≥70%), moderate risk (50–69%), or low risk (<50%) of bias, consistent with thresholds defined in previous systematic reviews [19]. Two reviewers performed assessments independently, with discrepancies resolved through consensus. Conference abstracts and the single cross-sectional observational study were not appraised due to limited methodological detail.

### 2.9. Data Synthesis

A descriptive synthesis was conducted to summarize genotype–phenotype correlations, variant classes and dominant clinical manifestations, regional patterns, diagnostic delays and pitfalls, therapeutic response patterns, natural history and long-term outcomes. Results were presented using descriptive tables summarizing molecular variants, clinical manifestations, therapeutic outcomes, and geographic distribution. Given the rarity of congenital CD59 deficiency and the predominantly case report/series nature of the available evidence, along with substantial clinical and methodological heterogeneity, quantitative meta-analysis was not carried out. Data were synthesized descriptively using counts, proportions, and summary measures where sufficient information was available. Denominators were restricted to patients for whom the relevant characteristic was reported and missing or unreported information was not assumed to be absent. Variant-level and phenotype-level patterns were explored descriptively without formal pooled effect estimates or inferential comparisons. Phenotypic classification was performed using hierarchical domain categorization with binary system-level involvement followed by subphenotypic tagging to capture overlapping manifestations.

### 2.10. Reporting Bias Assessment

Assessment of reporting bias was not feasible due to the rarity of the disease and predominance of case reports.

### 2.11. Certainty Assessment

The overall certainty of evidence was assessed narratively. Given the predominance of descriptive study designs, the overall level of evidence was assessed to be low.

## 3. Results

### 3.1. Search Results

**Figure 1:**
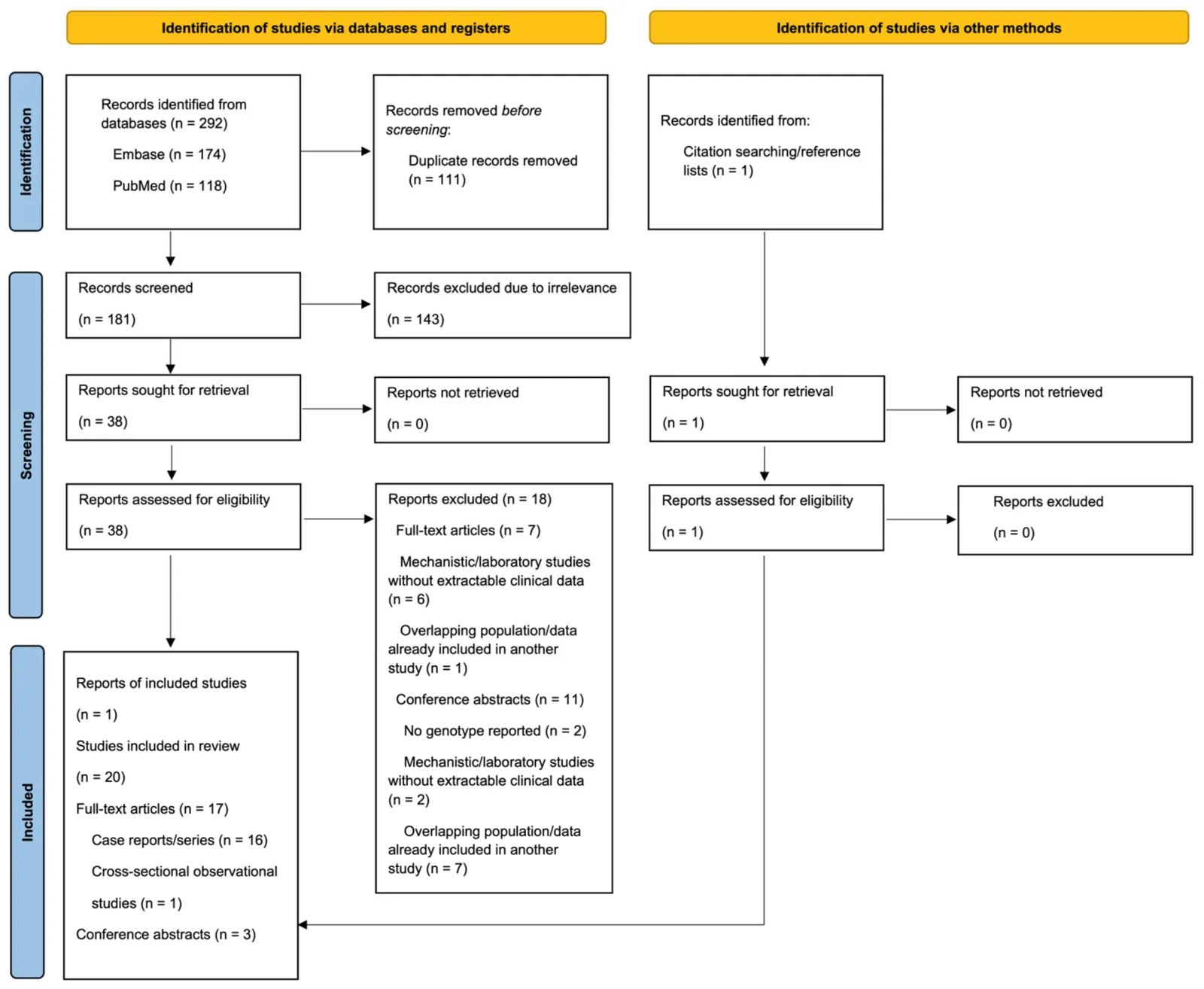
PRISMA flow chart illustrating the selection process. A total of 20 studies were included in this study.

The database search identified 292 records, including 174 from Embase and 118 from PubMed. After removal of 111 duplicate records, 181 records remained for title and abstract screening. During initial screening, 143 records were excluded due to irrelevance to congenital CD59 deficiency, leaving 38 reports for full eligibility assessment. All identified reports were retrieved. A total of 20 reports met inclusion criteria, consisting of 17 full-text articles, one of which was a cross-sectional observational study, and three conference abstracts. An additional report identified through other methods, Yamashina et al. [20], was found to describe the same patient reported by Motoyama et al. [21] and was therefore considered a duplicate report of the same case rather than a separate study. Accordingly, the total number of included studies remained 20. The study selection process is summarized in the PRISMA flow diagram (Figure 1).

### 3.2. Study Characteristics

A total of 20 studies were included in this review, consisting of 16 case reports/series, 1 cross-sectional study and 3 conference abstracts, as presented in Table 1. The primary synthesis included 31 patients reported in case reports/series. An additional 7 patients from cross-sectional studies and conference abstracts were identified but not included in the primary patient-level analyses due to insufficient reporting of the key elements. Among the case-based studies, 35.5% (11/31) were individual case reports, and 64.5% (20/31) were case series. Most studies were conducted in Turkey (n = 10), followed by Israel (n = 3), Germany (n = 2), Saudi Arabia (n = 1), USA (n = 1), Iran (n = 1), India (n = 1), and Japan (n = 1). Patient ages across case-based studies ranged from 2 months to 23 years, with both sexes represented. Ethnicity was most reported as Turkish, Libyan, Bahraini, Middle Eastern, Indian, Moroccan, or Egyptian. All case reports/series included molecular confirmation of the diagnosis. The molecular diagnostic methods reported varied between studies and included whole-exome sequencing (WES), targeted next-generation sequencing (NGS) gene panels, Sanger sequencing, polymerase chain reaction (PCR)-based assays, and targeted mutation analysis. Earlier reports more commonly relied on PCR-based assays, targeted mutation analysis, or Sanger sequencing, whereas more recent studies increasingly utilized WES and targeted NGS gene panels, particularly in larger contemporary cohorts such as that reported by Lateefa M. Almutawea (n = 8). The single cross-sectional study screened 71 patients, of whom 4 were genetically confirmed. These confirmed cases ranged in age from 2 to 10 years and included both males and females. Genetic confirmation was performed using WES and Sanger sequencing. Three conference abstracts were also included. Two described small case series (n = 2, n = 1), while one reported a larger clinical cohort (n = 15). To note, individual-level demographic and molecular diagnostic methods were inconsistently reported.

### 3.3. Risk of Bias

The methodological quality of the 16 included case-based studies was assessed using the Joanna Briggs Institute (JBI) critical appraisal tools. The detailed appraisal results are provided in Table S4. All 11 case reports were classified as low risk of bias, with total quality scores ranging from 75% to 100%. The overall reporting quality was high and considered sufficient for descriptive synthesis. Among the five included case series, all studies were classified as low risk of bias (80%), with one study graded with a score of 70%. Statistical analysis was deemed not applicable in all studies due to the descriptive design. No studies were excluded based on quality. Abstracts and cross-sectional observational studies lacked sufficient detail for full assessment.

### 3.4. Population Description

A total of 31 patients were identified across case reports and case series, with 7 additional patients from cross-sectional studies and abstracts summarized separately. The mean age at presentation was 5.05 years (range, 0.17–23 years; n = 29) (Table S5). Age at diagnosis was heterogeneously reported across the included studies. Eight patients were diagnosed in infancy, one in childhood, one during adolescence, and four postmortems. Exact numerical ages at diagnosis were available for two patients (3 and 6 years) while the remaining 15 patients were unreported. Disease onset was predominantly early in life, with infantile onset (<2 years) reported in 26/31 patients, including one patient with neonatal onset (<1 month). Onset timing was not reported in 2/31 patients. There was a slight female predominance, with 18/31 females and 13/31 males. Consanguinity status was available for 31 patients, of whom 18 were consanguineous. Of those with reported consanguinity, 16 cases had first-degree consanguinity, and two cases involved more remote consanguinity through shared grandparents. Family history data were available in 23 patients. Of these, 12 patients had a positive family history of affected relatives. The most affected relative was a sibling, accounting for 11 of 12 reported relatives. Additional reported affected relatives included nephews and extended maternal cousins.

### 3.5. Genotype

#### 3.5.1. Variant Distribution

All 31 patients harbored homozygous CD59 variants, confirming autosomal recessive inheritance. Eight unique pathogenic variants were identified (Table S6). Loss-of-function variants accounted for 18/31 cases. The most frequent variant was c.323C>A (10/31), followed by c.266G>A (7/31). The recurrent missense variant c.146A>T (p.Asp49Val) was identified in 5/31 cases. Together, three recurrent variants (c.323C>A, c.266G>A, and c.146A>T) accounted for 22/31 of the entire cohort, demonstrating marked mutational clustering and suggesting strong founder or regional effects.

#### 3.5.2. Founder Variant and Geographic Distribution

Founder variant pattern was identified in 10/31 patients, predominantly in Middle Eastern populations with eight and two cases in Bahrain and Israel respectively. Founder pattern was primarily associated with variants c.323C>A and c.266G>A. While ethnicity was previously summarized within study characteristics, the overall geographic distribution of reported patients shows a predominance in the Middle East and Mediterranean regions, with additional isolated cases from Europe, South Asia, and North America. However, the predominance of recurrent founder variants within specific Middle Eastern populations contrasts with the more genetically heterogeneous variant spectrum reported in other regions.

#### 3.5.3. Genotype Clustering, Mapping, and Association

CD59 variants demonstrated a non-random distribution with clear clustering within specific coding regions, as shown in Table 2. Exon 3 accounted for 11/31 of the total cohort and 11/15 of reported exons, representing the dominant mutational hotspot. In contrast, exon 5 and exon 4 contributed 2/31 and 1/31 of the total cohort respectively, while intron 1 variants were identified in 1/31 cases. Notably, exon data were unavailable in 16/31 cases. Multi-system involvement was present in 19/31 of the cohort. Across variant classes, missense variants demonstrated multi-system disease in 8/13, comparable to nonsense variants (6/10), while frameshift variants showed slightly lower involvement (3/6). The single reported splice-site variant, c.67+1G>T, was associated with severe and multisystem disease in the patient carrying this variant. Severe phenotype was observed in 21/31 of the cohort overall. Variant-specific findings showed heterogeneity, with c.266G>A and c.67+1G>T associated with severe phenotype in all reported patients. A severe phenotype was also observed in 3/5 patients with c.146A>T, 3/5 with c.146delA, and 6/10 with c.323C>A. In contrast, the single patient with c.286_295del did not present with a severe phenotype, and the single patient with c.85T>G did not meet the predefined criteria for severe phenotype. Exploratory stratification by functional consequence showed variation across the three functional groups. Predicted loss-of-function variants (nonsense, frameshift, and splice-site; n = 18) were associated with severe phenotype in 11/18 patients, compared with 7/7 patients with the domain-critical missense variant c.266G>A and 3/6 patients with other missense variants. These findings are descriptive and should be interpreted within the context of the small and unevenly represented variant groups and the predefined operational severity definition used in this review as they do not constitute validated measures of genotype-specific severity.

### 3.6. Phenotype

#### Dominant Phenotype Axis and Expanded Phenotypic Spectrum

CD59 deficiency was characterized predominately by a neuromuscular phenotype, representing the core disease axis across the cohort. The neuromuscular phenotype was highly penetrant, as shown in Table 3. Areflexia (22/31), muscle weakness (21/31), and hypotonia (20/31) were the most frequent features, followed by limb weakness (15/31), motor involvement (13/31), and limb paralysis (12/31). Chronic manifestations included chronic polyneuropathy (11/31) and muscle atrophy (11/31), while distal weakness and sensory involvement were less frequent, 5/31 each. The expanded phenotypic spectrum was characterized by CNS manifestations representing a secondary axis, with GBS (7/31) and stroke (7/31) as the most frequent presentations along with developmental delay (7/31) and quadriparesis (6/31). Additional features include dysphagia (4/31) and seizures, dysarthria, and optic neuritis (3/31 each). Less frequent neurological features (≤2/31) included cranial nerve involvement (LMN facial weakness, abducens palsy), cerebellar signs (ataxia, nystagmus), and severe inflammatory complications such as acute necrotizing encephalitis. Rare neurological complications (≤1/31) encompassed severe neuroinflammatory and vascular phenotypes including acute disseminated encephalomyelitis, transverse myelitis, dysmetria, dysesthesia, convulsions, myelitis, paresthesia, Moyamoya syndrome, and hemiparesis. A complement-mediated hemolytic phenotype was observed in over half of patients with hemolytic anemia in 18/31, hematuria 5/31, and hemolytic uremic syndrome in 2/31. Rare hematologic findings (≤1/31) included PNH-like features, systemic lupus erythematosus, and chronic anemia. Angioedema was reported in 5/31, reflecting broader systemic involvement. Musculoskeletal and structural abnormalities (e.g., pes deformities, scoliosis, contractures) were infrequent and may have reflected chronic neuromuscular involvement.

### 3.7. Disease Course

The disease course was characterized by a relapsing, trigger-dependent trajectory with high clinical variability. Infection-triggered exacerbations were the dominant precipitating factor reported (28/31) and a relapsing-remitting pattern was observed in 19/31 cases. Persistent or progressive disease without clear remission was less common. Documented relapse frequency was available for 20/31 cases, with a subset demonstrating reduced relapse frequency (9/20), often in the context of ongoing treatment. For trigger-specific variables, denominators reflect patients with available information. The analysis demonstrated a clear predominance of infectious stimuli, which frequently overlapped as they were reported heterogeneously, and individual patients could have more than one potential trigger associated with the same relapse. Vaccination or routine immunization was reported in temporal association with relapse in 10/31 patients. Other features included viral prodrome (8/31), intercurrent infection (6/31), febrile illness (5/31), and upper respiratory tract infection (5/31). Because these categories were not mutually exclusive, proportions do not sum to 100%. Vaccination/immunization was considered a temporal association only and was not evidence of causation, particularly because other potential concurrent triggers were reported in some of these cases. Response to immunomodulatory therapy was observed in 15/31 cases, supporting partial disease modulation despite the underlying genetic defect.

### 3.8. Outcomes and Survival

Outcome reflected a substantial disease burden. Among 31 patients, 22 were reported as alive at the last documented assessment, 8 as deceased, while survival status was not reported for one patient. Among the reported deaths, age at death ranged from 2.5 to 16 years (median, 9 years). Reported causes of death each included acute respiratory failure, pulmonary infection, septic shock, and severe cerebrovascular disease, although the cause of death was not reported in three of the deceased patients. Clinical outcome was reported as poor in 10/31, although this variable was not documented in 21/31, limiting definitive interpretation. Supportive care requirements were considerable. Mechanical ventilation was required in 11/31 patients, while 14/31 required blood transfusion, reflecting both neuromuscular respiratory compromise and hemolytic burden. No numerical follow-up duration was consistently reported. Therefore, the longest follow-up duration could not be reliably determined.

### 3.9. Diagnostic Patterns

Genetic testing represented the primary diagnostic method in the majority of cases, with a clear dominance of sequencing approaches. Targeted NGS panels, either alone or in combination with other methods, accounted for 14/31 of diagnoses, followed closely by WES at 11/31. Together, these approaches accounted for 25/31 genetic diagnoses. Targeted mutation analysis and PCR-based sequencing were less frequently utilized, representing 3/31 each.

Flow cytometry demonstrated a characteristic diagnostic signature, with complete absence of CD59 expression observed in 16/31 of cases. A PNH-like flow-cytometric phenotype was observed in 8/31 cases, reflecting overlapping complement-mediated hemolytic features associated with constitutional CD59 deficiency. A small subset (1/31) demonstrated residual CD59 expression (>1%), while 6/31 were not reported. Additionally, laboratory abnormalities demonstrated a consistent pattern dominated by biochemical evidence of hemolysis and neurochemical disturbance. Elevated CSF protein was the most frequent finding, present in 13/31 cases. Hemolytic markers were also prominent, with elevated LDH observed in 11/31 and reticulocytosis in 7/31, alongside a negative Coombs test in 6/31 of cases. Less common findings included CSF pleocytosis and isolated cytopenias, with thrombocytopenia and lymphopenia each reported in 1/31. Other abnormalities, including coagulation derangement, elevated liver enzymes, and renal impairment, were each identified in 1/31 cases, indicating systemic involvement.

Despite increasing use of advanced molecular diagnostics, delayed recognition remained evident in a subset of patients. Overall, delayed recognition was identified in 3/31 cases, reflecting persistent diagnostic challenges despite the emergence of advanced molecular tools. Stratification by variant class demonstrated heterogeneity in diagnostic timing. Early recognition was most frequent in missense variants (5/13) and frameshift variants (2/7), whereas nonsense variants were predominantly classified as indeterminate (8/10), suggesting diagnostic uncertainty rather than clear delay. The splice-site variant was uniformly associated with delayed recognition (1/1), although this reflects a single case. Missed diagnoses occurred across variant classes, most notably in frameshift (1/7) and missense variants (2/13), indicating that even structurally disruptive variants are not consistently identified early.

### 3.10. Treatment Response

Therapeutic strategies were heterogeneous and demonstrated clear patterns, as shown in Table 4. IVIG was the most administered therapy (24/31), followed by corticosteroids (19/31) and eculizumab (13/31), while plasma exchange was less frequently utilized (6/31). Treatment use differed across severity categories in which IVIG and corticosteroids were predominantly used in severe disease, with 18/24 and 14/19 cases respectively, while eculizumab showed a more balanced distribution between severe (6/13) and non-severe cases (7/13). Plasma exchange was more commonly reserved for severe presentations (4/6). Relapse remained frequent despite treatment, particularly with IVIG (14/24) and corticosteroids (11/19), while eculizumab demonstrated lower relapse rates (5/13). Notably, no deaths were reported among patients receiving eculizumab, compared to 1/24 receiving IVIG, 1/19 receiving corticosteroids, and 1/6 receiving plasma exchange. Among patients with reported responses, IVIG was associated predominantly with partial responses (19/23), while 3 patients achieved positive or dramatic responses, and 1 showed no response. Outcomes were not reported for 1 of the 24 treated patients. Corticosteroids demonstrated a mixed profile, with partial response in 12/17, positive response in 3/17, and no response in 2/17, with outcomes unreported for 2 of the 19 patients treated. Eculizumab showed a broader response distribution, with positive or dramatic responses in most patients (11/13), alongside 1 partial response and 1 non-response. Plasma exchange was associated exclusively with partial responses (5/5) among those with known outcomes, with 1 of the 6 treated patients lacking reported data.

To note, additional therapeutic modalities were observed in a subset of patients who required combination immunosuppressive strategies, including agents such as tacrolimus, mycophenolate mofetil, azathioprine, rituximab, cyclophosphamide, hydroxychloroquine, and adjunctive supportive therapies. Treatment initiation demonstrated wide variability, ranging from early infancy to later childhood, without a uniform pattern. Adverse events were reported across therapies with complications including hemolytic uremic syndrome-like episodes during plasma exchange, as well as neurologic and systemic effects with corticosteroids, including hypertension, seizures, and nystagmus. In addition, worsening encephalopathy and bulbar dysfunction (e.g., dysphagia) were observed in association with IVIG and corticosteroid therapy in some cases. Supportive care requirements further contextualize disease severity, with blood transfusion and mechanical ventilation as previously reported, often in conjunction with acute neurologic or hemolytic deterioration.

## 4. Discussion

### 4.1. Summary of Principal Findings

This systematic review provides a comprehensive synthesis of genetically confirmed congenital CD59 deficiency to date, integrating data across 31 patients reported. Despite identifying eight distinct pathogenic variants distributed across multiple variant classes, we observed a consistent neuro-hematologic phenotype, characterized predominantly by recurrent demyelinating neuropathy, Coombs-negative hemolytic anemia, and ischemic cerebrovascular events. Genotypic analysis demonstrated clustering of pathogenic variants within exon 3, which accounted for 11/15 of all reported exon-localized variants, suggesting the presence of a mutational hotspot despite the overall molecular heterogeneity of the disease. Exploratory stratification by predicted functional consequence suggested variation in severe phenotype across variant groups, with complete loss-of-function and domain-critical variants exhibiting the highest burden of severe multisystem disease. Therapeutically, although IVIG (24/31) and corticosteroids (19/31) remained the most frequently administered acute therapies, eculizumab (13/31) demonstrated the strongest biologic rationale as the only disease-directed treatment targeting terminal complement activation, despite heterogeneous clinical responses. Finally, the recurrent diagnostic delays observed across published cases, frequently following repeated presentations with GBS/CIDP-like neuropathy or unexplained Coombs-negative hemolysis, emphasize that delayed recognition remains one of the few potentially modifiable determinants of long-term neurological outcome.

### 4.2. CD59 Is a Distinct Neurohematologic Complement-Mediated Disorder

Congenital CD59 deficiency appears to represent a distinct neuro-hematologic disorder of complement dysregulation. Although it is caused by diverse pathogenic variants, the clinical phenotype observed across the included cases was remarkably reproducible. Rather than representing isolated organ-specific manifestations, these findings support the concept that congenital CD59 deficiency is a systemic complement-mediated disorder in which dysregulated terminal complement activation simultaneously affects peripheral nerves, erythrocytes, and the vascular endothelium. This observation is biologically consistent with the physiological role of CD59 as the principal inhibitor of MAC formation on host cell surfaces and with previous mechanistic studies demonstrating complement-mediated injury following loss of CD59 function [1,29,30,31].

The neurological presentation frequently mimics GBS or CIDP, explaining why many patients initially received repeated courses of IVIG, corticosteroids, or plasma exchange before the underlying diagnosis was recognized. However, unlike classical GBS, which is typically monophasic, or CIDP, which is primarily an immune-mediated peripheral neuropathy, congenital CD59 deficiency is characterized by recurrent demyelinating episodes occurring alongside complement-mediated hemolysis and, in many patients, recurrent ischemic cerebrovascular events [32,33,34]. The coexistence of these neurological, hematological, and vascular manifestations should therefore prompt consideration of an inherited complement regulatory defect rather than isolated inflammatory neuropathy, particularly in children with recurrent disease or those from consanguineous families. Positioning congenital CD59 deficiency within the broader spectrum of complement disorders further solidifies its distinctive clinicopathologic profile. Similar to PNH, both conditions share uncontrolled terminal complement activation and MAC-mediated hemolysis. However, PNH results from acquired somatic PIGA mutations causing loss of multiple GPI-anchored proteins, whereas congenital CD59 deficiency arises from inherited biallelic CD59 variants with preserved expression of other GPI-linked complement regulators [35,36]. Likewise, atypical hemolytic uremic syndrome (aHUS) is characterized by complement dysregulation and endothelial injury leading predominantly to thrombotic microangiopathy and renal involvement, whereas vascular manifestations in congenital CD59 deficiency are accompanied by prominent peripheral nervous system and hematologic disease [37]. In contrast, C3 glomerulopathy primarily reflects dysregulation of the alternative complement pathway with renal limited injury, emphasizing that congenital CD59 deficiency differs from many complement-mediated disorders by producing simultaneous neurological, hematological, and vascular involvement rather than predominantly single-organ disease [38]. Collectively, these observations support classifying congenital CD59 deficiency as a distinct multisystem neuro-hematologic disorder rather than a predominantly single-organ disease. Recognizing this convergence of the disease profile may facilitate earlier diagnostic evaluation and timely initiation of targeted complement inhibition before irreversible neurological injury occurs.

### 4.3. Functional Hierarchy

Missense variants represented the largest single mutational class in our cohort (13/31) though patients harboring missense changes displayed a wide spectrum of disease severity, from mild isolated neuropathy to infantile-onset severe multisystem disease. In contrast, truncating variants, the splice-site mutation c.67+1G>T, and the domain-critical missense variant c.266G>A were observed among patients with severe phenotypes. These findings suggest that consideration of the predicted functional effect and the affected protein domain may provide additional context for interpreting genotype–phenotype patterns beyond mutation class alone [39,40]. Based on variant consequence, location within the CD59 protein, and associated clinical phenotype, we explored a three-group functional framework (Table 2). Group A included variants predicted to cause complete loss of function, including the canonical splice-site mutation c.67+1G>T together with truncating nonsense and frameshift variants (e.g., c.146delA, c.286_295del). Group B comprised domain-critical missense variants, most notably c.266G>A (p.Cys89Tyr), which, despite being classified as missense, behaved phenotypically like a loss-of-function variant. Group C included peripheral missense variants (e.g., c.146A>T, c.85T>G) that were associated with milder phenotypes or later onset and variable reported functional effects. This functional hierarchy is consistent with previous experimental studies demonstrating that pathogenic CD59 variants impair complement inhibitory activity to varying degrees [5,9]. The c.266G>A (p.Cys89Tyr) variant provides a compelling example of why functional consequence outweighs variant nomenclature. Cysteine 89 is located within the extracellular Ly6/uPAR (LU) domain of CD59, which mediates interactions with complement components C8 and C9 and is essential for inhibition of MAC assembly [41,42]. Structural and mutagenesis studies have identified specific CD59 residues within this domain that contribute directly to the C8/C9-binding interface and demonstrated that disruption of this region can impair CD59-mediated inhibition of MAC assembly [43,44]. Consistent with these mechanistic findings, all patients in our review carrying c.266G>A exhibited severe early-onset disease with recurrent hemolysis and neurological involvement, a phenotype closely resembling that was observed with truncating variants. Although the present findings cannot establish a validated genotype–phenotype relationship, the observed pattern raises the hypothesis that predicted functional consequence and structural location may provide greater insight into phenotypic severity. This hypothesis requires confirmation in larger cohorts with standardized phenotyping and functional characterization.

Structural mapping of the cohort variants provides additional mechanistic context for this functional hierarchy. The experimentally characterized CD59-C8α interface includes key residues F47, E58, Y61, and Y62, while CD59 also interacts with the extending C9 β-hairpins through distinct surface loops to prevent membrane insertion [43,44]. In our cohort, c.146delA (p.Asp49Valfs*31) occurs immediately downstream of F47 and may disrupt the surrounding C8-binding region, whereas c.146A>T (p.Asp49Val) affects the same structural neighborhood and has been associated with loss of surface CD59. The c.85T>G (p.Tyr29Asp) variant lies near the CD59 32–34 loop implicated in the C9 interaction, although its functional and clinical significance remains uncertain given the single reported case [44]. In contrast, truncating and splice-site variants such as c.67+1G>T, c.286_295del, c.323C>A, and c.123del (p.Val42fs) are more appropriately interpreted as causing loss of functional CD59 rather than disruption of a specific C8/C9 contact residue. These assignments are mechanistic interpretations based on established CD59 structure-function data and should not be considered direct functional validation of the individual variants.

Exon 3 emerged as a mutational hotspot, accounting for 11/15 of exon-localized variants identified in this review. Exon 3 encodes a substantial portion of the extracellular Ly6/uPAR domain of CD59, which mediates binding to complement components C8 and C9 and inhibits MAC formation [42,44]. Missense variants clustering within this domain may be more likely to disrupt MAC-binding and compromise complement inhibition than variants located in peripheral regions or the signal peptide. The observed exon 3 clustering is consistent with enrichment of variants within the extracellular complement inhibitory domain, although the available data do not establish that exon location alone determines functional severity. The integration of these data supports a biologic model in which the degree of disruption of CD59 structure and function may determine the intensity of terminal complement activation and, consequently, the clinical phenotype. Variants that abolish CD59 expression or severely disrupt the C8/C9-binding interface may result in uncontrolled MAC deposition on erythrocytes, leukocytes, endothelium, and peripheral nerves, resulting in Coombs-negative hemolytic anemia, demyelinating neuropathy, and ischemic stroke. Variants with partial functional impairment may allow residual complement regulation, producing milder or later-onset disease [5,9]. This proposed hierarchical gradient of functional disruption is provided in Table S7.

### 4.4. Diagnostic Delays

Despite the reproducible clinical phenotype observed across our cohort, congenital CD59 deficiency remained consistently underrecognized, resulting in substantial diagnostic delay. This delay was frequently described in reported cases, particularly among patients initially presenting with recurrent demyelinating neuropathy or GBS/CIDP-like phenotypes. Exact ages at symptom onset and diagnosis were inconsistently reported across literature, precluding reliable quantitative estimation of diagnostic delay across the cohort. During this interval, patients frequently experienced recurrent neurological attacks, hemolytic episodes, and cerebrovascular events before the underlying complement disorder was recognized. Similar diagnostic challenges have been reported, with prolonged diagnostic intervals preceding irreversible neurological impairment despite characteristic manifestations from infancy [9,17,45].

**Figure 2:**
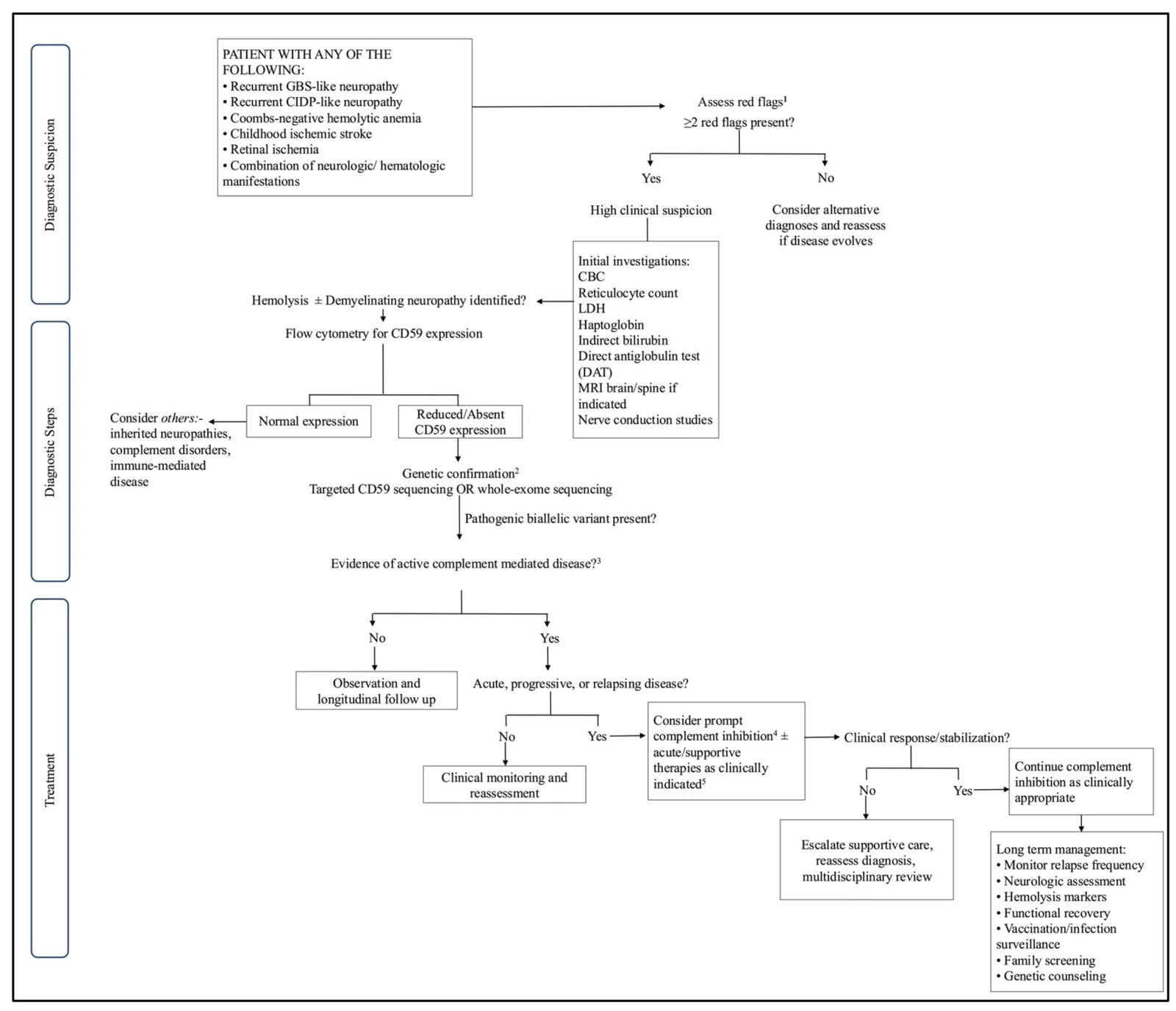
Proposed Diagnostic and Management Algorithm for Suspected Congenital CD59 Deficiency. ^1^ Clinical suspicion should be heightened in patients presenting with two or more listed characteristic features. Infantile disease onset, parental consanguinity, recurrent relapses, affected siblings, and incomplete response to conventional immunotherapy further increase the likelihood of congenital CD59 deficiency. ^2^ Segregation analysis should be considered where feasible to support variant interpretation and facilitate family counseling. ^3^ Active disease refers to ongoing complement-mediated tissue injury rather than historical disease severity and includes recurrent demyelinating neuropathy, persistent hemolysis, progressive neurological deterioration, recurrent cerebrovascular events, or repeated disease relapses. ^4^ Early initiation before irreversible neurological injury may maximize clinical benefit. Complement inhibition is a mechanism-directed therapeutic consideration based on limited clinical experience and should be individualized according to disease activity, severity, and recurrence, with vaccination and appropriate antimicrobial prophylaxis addressed according to established recommendations for C5 inhibitor therapy. ^5^ Acute therapy modalities frequently produced transient or partial neurological improvement with no prevention of subsequent relapses or long-term disease progression.

The observed diagnostic delay appears largely attributable to phenotypic overlap with more common inflammatory neuropathies. Most patients initially presented with recurrent GBS/CIDP-like neuropathy and consequently received repeated courses of IVIG, corticosteroids, or plasma exchange before definitive diagnosis [46,47]. Although these therapies often produced transient neurological improvement, relapses remained common because the underlying mechanism of uncontrolled terminal complement activation persisted [48,49]. Recognition of this characteristic neurohematologic constellation, particularly in infants and young children, should therefore prompt investigation for inherited complement dysregulation rather than isolated autoimmune neuropathy [9,19]. Our findings further support the incorporation of flow cytometric assessment of CD59 expression as an early screening investigation, followed by confirmatory molecular testing [2,9]. Based on these findings, we propose a clinical diagnostic algorithm (Figure 2) to facilitate earlier recognition and prompt initiation of complement-directed therapy. This algorithm represents expert opinion derived from the available published evidence, which consists predominantly of uncontrolled case reports/series, and has not been prospectively validated. Given the superior biological rationale of terminal complement inhibition compared with conventional immunomodulatory therapies, our findings suggest that early recognition may be one of the most effective strategies for potentially preventing irreversible neurological injury and supporting better long-term outcomes in congenital CD59 deficiency [48,49,50].

### 4.5. Therapeutic Implications

Unlike conventional immunomodulatory therapies, which primarily suppress secondary inflammatory responses, eculizumab directly targets the underlying disease mechanism by preventing cleavage of complement component C5 and subsequent MAC formation [51]. The clinical experience with eculizumab across our cohort supports its role as a mechanism-directed treatment, although responses remain heterogeneous. In contrast, the limited durability of responses reported with conventional immunomodulatory therapies, e.g., IVIG, corticosteroids, and plasma exchange, is consistent with previous reports demonstrating that complement blockade, rather than immunosuppression alone, is required to interrupt the ongoing complement-mediated tissue injury and improve disease control [5,9,15]. These combination immunosuppressive approaches were primarily utilized in cases with persistent or relapsing disease, where single-modality treatment failed to achieve sustained control. Importantly, the reported adverse events often overlapped with severe disease activity and relapse episodes, making attribution solely to therapy effects or the underlying disease challenging. Overall, the observed pattern suggests that treatment-related complications tend to cluster in patients with high disease burden rather than representing isolated drug toxicity.

Nevertheless, treatment outcomes remained heterogeneous, with some patients continuing to experience relapses or limited neurological recovery despite complement inhibition. Several factors may explain this variability. First, irreversible axonal injury and chronic neurodegeneration may already be established by the time eculizumab is initiated, limiting the potential for neurological recovery despite effective complement blockade [15]. Second, differences in residual CD59 function may potentially influence therapeutic responsiveness, particularly among variants associated with complete loss of function compared with those retaining partial complement inhibitory activity [5,9]. Third, prolonged disease duration before treatment initiation likely contributes to cumulative neurological damage, while publication bias toward successful therapeutic outcomes cannot be excluded given the predominance of case reports and small case series. Collectively, these findings suggest that the timing of complement inhibition may be as important as treatment selection itself [5,9,15].

### 4.6. Future Directions

Despite the severe clinical course observed in many affected individuals, survival into adolescence is possible. However, heterogeneous and incomplete follow-up limits the ability to define long-term prognosis or life expectancy. Longer-term prospective follow-up will therefore be required to better define the natural history and survival of affected individuals. The predominance of congenital CD59 deficiency in consanguineous populations, particularly within the Gulf region, supports targeted screening in high-risk families through earlier genetic testing, cascade family screening, premarital counseling, and targeted sequencing of recurrent regional variants [6]. Current evidence remains limited to isolated case reports and small case series, highlighting the need for an international patient registry to better define the natural history, long-term outcomes, and predictors of treatment response. Future studies should also validate the proposed functional genotype–phenotype classification through mechanistic and structural analyses and identify biomarkers such as residual CD59 expression and complement activity to improve prognostic stratification. Given the monogenic nature of the disease and advances in gene-editing technologies, gene replacement or gene-editing strategies may represent future therapeutic avenues [52,53].

### 4.7. Strengths and Limitations

To our knowledge, this represents the largest synthesis of reported patients with CD59 deficiency to date. However, the available evidence remains limited to 31 heterogeneously reported patients from case-based studies. Major strengths include comprehensive genotype–phenotype analysis, aggregation of treatment outcomes, evaluation of regional and founder variant patterns. Nevertheless, several limitations should be acknowledged. The available evidence consisted predominantly of case reports and small case series, increasing susceptibility to publication bias, incomplete reporting, heterogeneous follow-up, and limited generalizability. The rarity of the disease and small sample size also precluded robust statistical inference and causal assessment of treatment effects.

## 5. Conclusions

This systematic review synthesized the available evidence on the genetic architecture, clinical spectrum, diagnostic approaches, therapeutic outcomes, and geographic distribution of congenital CD59 deficiency. The findings expand the current understanding of the phenotypic and molecular heterogeneity of this rare complement-mediated disorder and reflect the persistent challenges in diagnosis and management. Future collaborative studies, standardized reporting, and international patient registries are needed to strengthen the evidence base, and the exploratory genotype–phenotype findings require validation in larger cohorts with standardized phenotyping to guide the development of diagnostic and therapeutic strategies for congenital CD59 deficiency.

## Figures and Tables

**Table 1:** Summary of Included Studies.

Case Reports/Series							
Author, Year	Country	Study Design	Number of Cases	Ethnicity	Age	Gender	Molecular Diagnostic Method
Chai et al. 2022 [1]	USA	Case report	1	Middle Eastern	5 years	Male	Targeted NGS panel
Cornelius et al. 2025 [4]	India	Case report	1	Indian	4.5 years	Male	WES
Almutawe et al. 2023 [5]	Bahrain	Case series	8	Bahraini	10 months	Female	Targeted NGS panel
					11 months	Female	Targeted NGS panel
					11 months	Male	Targeted NGS panel
					2 months	Female	Targeted NGS panel
					4 months	Female	Targeted NGS panel
					5 months	Female	Targeted NGS panel
					6 months	Female	Targeted NGS panel
					11 months	Male	Targeted NGS panel
Nevo et al. 2012 [9]	Israel	Case series	5	Libyan/Moroccan	13 months	Male	Targeted mutation analysis
				Libyan	18 months	Female	WES
				Libyan	3.5 years	Male	WES
				Moroccan/Egyptian	54 months	Male	Targeted mutation analysis
				Libyan	55 months	Female	Targeted mutation analysis
Ardicli et al. 2017 [10]	Turkey	Case report	1	Not reported	7 years	Female	WES
Ben-Zeev et al. 2015 [11]	Israel	Case series	2	Libyan	2.5 years	Female	PCR of stored muscle biopsy DNA
				Libyan	8 years	Male	PCR of stored muscle biopsy DNA
Günay et al. 2022 [12]	Turkey	Case report	1	Not reported	20 months	Female	Sanger sequencing
Klemann et al. 2018 [13]	Germany	Case report	1	Turkish	6 years	Male	Sanger sequencing
Höchsmann et al. 2014 [15]	Germany	Case report	1	Turkish	60 months	Female	DNA sequencing of granulocytes and oral mucosa
Yamashina et al. 1990 [20] Motoyama et al. 1992 [21]	Japan	Case report	1	Not reported	23 years	Male	PCR amplification Sanger sequencing Genomic DNA from peripheral blood lymphocytes
Yuksel et al. 2018 [22]	Turkey	Case series	2	Turkish	10 years	Male	Targeted NGS panel
				Turkish	11 years	Female	Targeted NGS panel
Busaleh et al. 2025 [23]	Saudi Arabia	Case report	1	Not reported	2 years	Female	WES
Güngör et al. 2022 [24]	Turkey	Case report	1	Turkish	2.5 years	Female	WES
Solmaz et al. 2020 [25]	Turkey	Case report	1	Turkish	6 years	Male	Targeted NGS panel Sanger sequencing
Javadi Parvaneh et al. 2020 [26]	Iran	Case report	1	Not reported	16 years	Female	Targeted NGS panel Sanger sequencing
Haliloglu et al. 2015 [27]	Turkey	Case series	3	Turkish	16 years	Female	WES Sanger sequencing
				Turkish	Not reported	Female	WES Sanger sequencing
				Turkish	Not reported	Male	WES Sanger sequencing
Observational Studies							
Sefika Ilknur et al. 2025 [6]	Turkey	Cross-sectional observational study	71 screened (4 confirmed)	Turkish	10 years	Female	WES Sanger sequencing
				Turkish	2 years	Male	WES Sanger sequencing
				Turkish	4 years	Male	WES Sanger sequencing
				Turkish	7 years	Female	WES Sanger sequencing
Conference Abstracts Reporting Cases or Case Series							
Mevorach 2016 [7]	Israel	Clinical study	15	Not reported	Not reported	Not reported	Not reported
Koca et al. 2016 [14]	Turkey	Case report	1	Not reported	11 years	Female	Not reported
Gumus et al. 2019 [28]	Turkey	Case series	2	Not reported	11 years	Male	Not reported
				Not reported	14 years	Female	Not reported

PCR: polymerase chain reaction; NGS: next-generation sequencing; WES: whole-exome sequencing.

**Table 2:** Genotype–Phenotype Characteristics of Congenital CD59 Deficiency.

Genotype Category	Specific Variant/ Exon	n/N of Cohort	Functional Association	Multisystem Involvement n/N ^†^	Severe Phenotype n/N *
Exon distribution	Exon 3	11/31	Predominant exon involved	–	–
	Exon 5	2/31	Less frequent	–	–
	Exon 4	1/31	Rare	–	–
	Intron 1	1/31	Non-coding variant	–	–
	Not reported	16/31	–	–	–
Variant type	Missense	13/31	Variable functional impact	8/13	–
	Nonsense	10/31	Truncating variants	6/10	–
	Frameshift	7/31	Loss-of-function spectrum	4/7	–
	Splice-site	1/31	RNA splicing disruption	1/1	–
Variant-specific outcomes	c.266G>A	7/31	Exon 3 hotspot variant	–	7/7
	c.67+1G>T	1/31	Splice-site variant	–	1/1
	c.286_295del	1/31	Deletion variant	–	0/1
	c.146delA	5/31	Frameshift variant	–	3/5
	c.323C>A	10/31	Nonsense variant	–	6/10
	c.146A>T	5/31	Missense variant	–	3/5
	c.85T>G	1/31	Missense variant	–	0/1
	c.123del	1/31	Frameshift variant	–	1/1
Functional grouping	A: Complete loss (nonsense/frameshift/splice)	18/31	Loss-of-function	–	11/18
	B: Domain-critical missense	7/31	Exon 3 hotspot (c.266G>A)	–	7/7
	C: Other missense	6/31	Peripheral missense variants (c.146A>T, c.85T>G)	–	3/6

^†^ Multisystem involvement was defined as the presence of disease affecting more than one of the following systems/domains: hematologic, central or peripheral nervous system. * No validated severity classification currently exists for congenital CD59 deficiency. Therefore, severe phenotype was operationally post hoc for this review. This investigator-defined classification was applied retrospectively to heterogeneous published case descriptions. Specifically, severe phenotype was defined by the presence of ≥1 major organ-threatening manifestation (recurrent demyelinating neuropathy, central nervous system involvement, or transfusion-dependent hemolytic anemia), consistent with features associated with the greatest morbidity in published reports [5,9]. The resulting genotype-specific percentages are strictly descriptive of this literature cohort and are not interpreted as validated estimates of clinical risk.

**Table 3:** Phenotypic spectrum of congenital CD59 deficiency.

System	Reported Clinical Manifestations	n/N *
Neuromuscular	Areflexia	22/31
	Muscle weakness	21/31
	Hypotonia	20/31
	Limb weakness	15/31
	Motor involvement	13/31
	Limb paralysis	12/31
	Chronic polyneuropathy	11/31
	Muscle atrophy	11/31
	Hand/foot weakness	5/31
	Sensory involvement	5/31
Neurologic	GBS	7/31
	Developmental delay	7/31
	Stroke	7/31
	Quadriparesis	6/31
	Dysphagia	4/31
	Seizures	3/31
	Dysarthria	3/31
	Optic neuritis	3/31
Hematologic	Hemolytic anemia	18/31
	Hematuria	5/31
	Hemolytic uremic syndrome	2/31
	Mild anemia	2/31
Systemic	Angioedema	5/31
	Apathy	2/31
Musculoskeletal	Ankle contractures	2/31
	Thoracolumbar scoliosis	2/31
	Equinovarus deformity	2/31
	Pes cavus	2/31
	Pes planus	1/31
	Hypertonia	1/31

* Clinical manifestations were extracted as reported in the original publications and organized by clinical system. Related manifestations may overlap within individual patients and are not mutually exclusive; frequencies should therefore not be summed to derive composite phenotype estimates.

**Table 4:** Treatment patterns and outcomes in congenital CD59 deficiency.

Therapy	Severity Markers				Response			
Type	n/N Used	Severe Disease n/N	Non-Severe n/N	Relapse n/N	Mortality n/N	None n/N	Partial Response n/N	Positive/Dramatic Response n/N
Eculizumab	13/31	6/13	7/13	5/13	0/13	1/13	1/13	11/13
IVIG	24/31	18/24	6/24	14/24	1/24	1/23	19/23	3/23
Corticosteroids	19/31	14/19	5/19	11/19	1/19	2/17	12/17	3/17
Plasma exchange	6/31	4/6	2/6	3/6	1/6	0/5	5/5	0/5

## Data Availability

The datasets of the manuscript are available from the corresponding author upon request.

## Supplementary Materials

The following supporting information can be downloaded at: https://www.mdpi.com/article/10.3390/jcm15197751/s1, Table S1: Clinical Phenotype and Reported Variants of Congenital CD59 Deficiency; Table S2: PRISMA 2020 Checklist; Table S3: Search strategy; Table S4: Risk Assessment of Included Case reports and Series; Table S5: Demographic Characteristics of Included Patients; Table S6: CD59 Variant Class and Distribution (n = 31); Table S7: Proposed Functional Framework for Interpreting Genotype-Phenotype Relationships in Congenital CD59 Deficiency.
