# Supplementary material for: Congenital/Inherited CD59 Deficiency: A Systematic Review
Source: J Clin Med. 2026 Oct 8;15(19):7751. doi: 10.3390/jcm15197751 (PMC13666367; doi:10.3390/jcm15197751)
Supplement: Supplementary file 1 [file jcm-15-07751-s001.zip › jcm-4478456-supplementary.pdf]

Table S1: Clinical Phenotype and Reported Variants of Congenital CD59 Deficiency

Domain	System	Feature
**Clinical Phenotype**	Muscle, Soft Tissue	Hypotonia
		Muscle weakness, upper and lower limbs
		Muscle atrophy, progressive
		Hand and foot weakness
	Neurologic<br>*Peripheral Nervous System*	Chronic immune-mediated polyneuropathy
		Limb weakness
		Limb paralysis
		Areflexia
		Sensory and motor demyelination seen on sural nerve biopsy
		Secondary axonal damage
	Hematology	Hemolytic anemia
	Laboratory Abnormalities	Increased CSF protein
		Absence of CD59 expression on red cells
	Miscellaneous	Onset in infancy (3 to 7 months)
		Exacerbations during infection
		Relapsing-remitting course
		Immunosuppressive therapy may be beneficial
**Reported Variants**	Missense	c.266G>A (p.Cys89Tyr)
		c.190G>A (p.Cys64Tyr)
		c.190T>G (p.Cys64Gly)
		c.83G>A (p.Cys28Tyr)
		c.146A>T (p.Asp49Val)
		c.71G>T (p.Asp24Val)
		c.85T>G (p.Tyr29Asp)
	Start-loss, missense	c.2T>C (p.Met1Thr)
	Nonsense, truncating	c.323C>A (p.Ser108Ter)
	Frameshift	c.71_72del (p.Asp24Valfs*)
		c.46_47del (p.Ala16Alafs*)
		c.146del (p.Asp49fs)
		c.286_295del (p.Phe96SerfsTer18)
		c.123delC (p.Val42Serfs*38)
		c.301del (p.Glu101fs)
	Duplication, frameshift	c.183_184dup (p.Asn62fs)
	Deletion, truncating	c.282del (p.Leu93_Cys94insTer)
		c.143_144del (p.Asp47Phefs*)
	Splice-site	c.67+1G>T

Table S2: PRISMA 2020 Checklist

Section and Topic	Item #	Checklist item	Location where item is reported
**TITLE**			
Title	1	Identify the report as a systematic review.	Page 1
**ABSTRACT**			
Abstract	2	See the PRISMA 2020 for Abstracts checklist.	Page 1
**INTRODUCTION**			
Rationale	3	Describe the rationale for the review in the context of existing knowledge.	Page 2
Objectives	4	Provide an explicit statement of the objective(s) or question(s) the review addresses.	Page 3
**METHODS**			
Eligibility criteria	5	Specify the inclusion and exclusion criteria for the review and how studies were grouped for the syntheses.	Page 3
Information sources	6	Specify all databases, registers, websites, organisations, reference lists and other sources searched or consulted to identify studies. Specify the date when each source was last searched or consulted.	Page 3
Search strategy	7	Present the full search strategies for all databases, registers and websites, including any filters and limits used.	Page 3
Selection process	8	Specify the methods used to decide whether a study met the inclusion criteria of the review, including how many reviewers screened each record and each report retrieved, whether they worked independently, and if applicable, details of automation tools used in the process.	Page 3
Data collection process	9	Specify the methods used to collect data from reports, including how many reviewers collected data from each report, whether they worked independently, any processes for obtaining or confirming data from study investigators, and if applicable, details of automation tools used in the process.	Page 4
Data items	10a	List and define all outcomes for which data were sought. Specify whether all results that were compatible with each outcome domain in each study were sought (e.g. for all measures, time points, analyses), and if not, the methods used to decide which results to collect.	Page 4
	10b	List and define all other variables for which data were sought (e.g. participant and intervention characteristics, funding sources). Describe any assumptions made about any missing or unclear information.	Page 4
Study risk of bias assessment	11	Specify the methods used to assess risk of bias in the included studies, including details of the tool(s) used, how many reviewers assessed each study and whether they worked independently, and if applicable, details of automation tools used in the process.	Page 4
Effect measures	12	Specify for each outcome the effect measure(s) (e.g. risk ratio, mean difference) used in the synthesis or presentation of results.	Not applicable
Synthesis methods	13a	Describe the processes used to decide which studies were eligible for each synthesis (e.g. tabulating the study intervention characteristics and comparing against the planned groups for each synthesis (item #5)).	Page 5
	13b	Describe any methods required to prepare the data for presentation or synthesis, such as handling of missing summary statistics, or data conversions.	Page 5
	13c	Describe any methods used to tabulate or visually display results of individual studies and syntheses.	Page 5
	13d	Describe any methods used to synthesize results and provide a rationale for the choice(s). If meta-analysis was performed, describe the model(s), method(s) to identify the presence and extent of statistical heterogeneity, and software package(s) used.	Page 5
	13e	Describe any methods used to explore possible causes of heterogeneity among study results (e.g. subgroup analysis, meta-regression).	Page 5
	13f	Describe any sensitivity analyses conducted to assess robustness of the synthesized results.	Not applicable
Reporting bias assessment	14	Describe any methods used to assess risk of bias due to missing results in a synthesis (arising from reporting biases).	Page 5
Certainty assessment	15	Describe any methods used to assess certainty (or confidence) in the body of evidence for an outcome.	Page 5

**RESULTS**			
Study selection	16a	Describe the results of the search and selection process, from the number of records identified in the search to the number of studies included in the review, ideally using a flow diagram.	Page 6
	16b	Cite studies that might appear to meet the inclusion criteria, but which were excluded, and explain why they were excluded.	Page 6
Study characteristics	17	Cite each included study and present its characteristics.	Page 7
Risk of bias in studies	18	Present assessments of risk of bias for each included study.	Page 8
Results of individual studies	19	For all outcomes, present, for each study: (a) summary statistics for each group (where appropriate) and (b) an effect estimate and its precision (e.g. confidence/credible interval), ideally using structured tables or plots.	Page 8
Results of syntheses	20a	For each synthesis, briefly summarise the characteristics and risk of bias among contributing studies.	Page 8-13
	20b	Present results of all statistical syntheses conducted. If meta-analysis was done, present for each the summary estimate and its precision (e.g. confidence/credible interval) and measures of statistical heterogeneity. If comparing groups, describe the direction of the effect.	Not applicable
	20c	Present results of all investigations of possible causes of heterogeneity among study results.	Page 8-13
	20d	Present results of all sensitivity analyses conducted to assess the robustness of the synthesized results.	Not applicable
Reporting biases	21	Present assessments of risk of bias due to missing results (arising from reporting biases) for each synthesis assessed.	Not applicable
Certainty of evidence	22	Present assessments of certainty (or confidence) in the body of evidence for each outcome assessed.	Not applicable
**DISCUSSION**			
Discussion	23a	Provide a general interpretation of the results in the context of other evidence.	Page 14-19
	23b	Discuss any limitations of the evidence included in the review.	Page 25
	23c	Discuss any limitations of the review processes used.	Page 25
	23d	Discuss implications of the results for practice, policy, and future research.	Page 24
**OTHER INFORMATION**			
Registration and protocol	24a	Provide registration information for the review, including register name and registration number, or state that the review was not registered.	Page 20
	24b	Indicate where the review protocol can be accessed, or state that a protocol was not prepared.	Page 19
	24c	Describe and explain any amendments to information provided at registration or in the protocol.	Not applicable
Support	25	Describe sources of financial or non-financial support for the review, and the role of the funders or sponsors in the review.	Page 19
Competing interests	26	Declare any competing interests of review authors.	Page 19
Availability of data, code and other materials	27	Report which of the following are publicly available and where they can be found: template data collection forms; data extracted from included studies; data used for all analyses; analytic code; any other materials used in the review.	Page 19

Table S3: Search strategy

**Pubmed/MEDLINE**	("CD59"[Mesh] OR "CD59 deficiency" OR "congenital CD59 deficiency"<br>OR "inherited CD59 deficiency")<br>AND<br>(deficiency OR mutation OR variant OR genetic OR gene OR congenital)
**Embase**	('cd59'/exp OR 'cd59 deficiency' OR 'congenital cd59 deficiency'<br>OR 'inherited cd59 deficiency')<br>AND<br>(deficiency OR mutation OR variant OR genetic OR gene OR congenital)

Table S4: Risk Assessment of Included Case reports and Series.

Case Reports										
**Author, Year**	**Domain**								**Yes (%)**	**Risk of Bias**
	Were patient's demographic characteristics clearly described?	Was the patient's history clearly described and presented as a timeline?	Was the current clinical condition of the patient on presentation clearly described?	Were diagnostic tests or assessment methods and the results clearly described?	Was the intervention(s) or treatment procedure(s) clearly described?	Was the post-intervention clinical condition clearly described?	Were adverse events (harms) or unanticipated events identified and described?	Does the case report provide takeaway lessons?		
Chai et al. 2022[1]	Yes	Yes	Yes	Yes	Yes	Yes	Yes	Yes	100	Low
Cornelius et al. 2025[4]	Yes	Yes	Yes	Yes	Yes	Yes	Yes	Yes	100	Low
Ardicli et al. 2017[10]	Yes	Yes	Yes	Yes	Yes	Yes	Yes	Yes	100	Low
Günay et al. 2022[12]	Yes	Yes	Yes	Yes	Yes	Yes	Yes	Yes	100	Low
Klemann et al. 2018[13]	Yes	Yes	Yes	Yes	Yes	Yes	No	Yes	87.5	Low
Höchsmann et al. 2014[15]	Yes	Yes	Yes	Yes	Yes	Yes	No	Yes	87.5	Low
Yamashina et al. 1990[20] Motoyama et al. 1992[21]	Yes	Yes	Yes	Yes	No	No	Yes	Yes	75	Low
Busaleh et al. 2025[23]	Yes	Yes	Yes	Yes	Yes	Yes	Unclear	Yes	87.5	Low
Güngör et al. 2022[24]	Yes	Yes	Yes	Yes	Yes	Yes	Yes	Yes	100	Low
Solmaz et al. 2020[25]	Yes	Yes	Yes	Yes	Yes	Yes	Yes	Yes	100	Low
Javadi Parvaneh et al. 2020[26]	Yes	Yes	Yes	Yes	Yes	Yes	No	Yes	87.5	Low

Case Series												
**Author, Year**	**Domain**										**Yes (%)**	**Risk of Bias**
	Were there clear criteria for inclusion in the case series?	Was the condition measured in a standard, reliable way for all participants included in the case series?	Were valid methods used for identification of the condition for all participants included in the case series?	Did the case series have consecutive inclusion of participants?	Did the case series have complete inclusion of participants?	Was there clear reporting of the demographics of the participants in the study?	Was there clear reporting of clinical information of the participants?	Were the outcomes or follow-up results of cases clearly reported?	Was there clear reporting of the presenting site(s)/clinic (s) demographic information?	Was statistical analysis appropriate?		
Almutawe et al. 2023[5]	Yes	Yes	Yes	Unclear	Yes	Yes	Yes	Yes	Yes	Not applicable	80	Low
Nevo et al. 2012[9]	Yes	Yes	Yes	No	Yes	Yes	Yes	Yes	Yes	Not applicable	80	Low
Ben-Zeev et al. 2015[11]	Yes	Yes	Yes	No	Yes	No	Yes	Yes	Yes	Not applicable	70	Moderate
Yuksel et al. 2018[22]	Yes	Yes	Yes	No	Yes	Yes	Yes	Yes	Yes	Not applicable	80	Low
Haliloglu et al. 2015[27]	Yes	Yes	Yes	No	Yes	Yes	Yes	Yes	Yes	Not applicable	80	Low

Table S5: Demographic Characteristics of Included Patients.

Variable	Overall
Age at presentation*, mean (range)	5.05 years (0.17-23 years)
Age at diagnosis*, mean	4.5 years
Male, n/N	13/31
Female, n/N	18/31
Consanguinity, n/N	18/31
Positive family history, n/N	12/23†
Most common affected relative, n/N	Sibling, 11/12

*Exact numeric age at presentation was available for 29 patients. Exact numeric age at diagnosis was available for 2 patients. A pooled quantitative estimate of diagnostic delay was not calculated due to incomplete reporting of exact onset and diagnosis ages.

†Family history reported in 23 patients.

Table S6: CD59 Variant Class and Distribution (n = 31).

Variable	Overall
Unique variants identified, n	8
Homozygous variants, n	31
Loss-of-function variants n/N*	18/31
c.323C>A	10/18
c.146delA	5/18
c.286_295del	1/18
c.67+1G>T	1/18
c.123del	1/18
Missense variants n/N	13/31
c.266G>A	7/13
c.146A>T	5/13
c.85T>G	1/13
Most common variant	c.323C>A
Second most common variant	c.266G>A

*Loss-of-function includes nonsense, frameshift and splice-site variants.

Table S7: Proposed Functional Framework for Interpreting Genotype-Phenotype Relationships in Congenital CD59 Deficiency.

Integrated Mechanistic Pathway	Proposed Functional Interpretation
**Exon 3 clustering**<br>→ Structural MAC-interface disruption<br>→ Complete loss of complement inhibition<br>→ Uncontrolled MAC deposition<br>→ Hemolysis + neuroinflammation<br>→ Severe infantile phenotype	**Highest predicted functional disruption**<br>Splice-site (c.67+1G>T)<br>↓<br>Domain-critical missense (c.266G>A)<br>↓<br>Frameshift variants (c.146delA, c.286_295del)<br>↓<br>Nonsense (c.323C>A)<br>↓<br>Peripheral missense (c.146A>T, c.85T>G)<br>**Lowest predicted disruption**
